# Intra-Leaf Variability of Incubation Period Sheds New Light on the Lifestyle of *Cercospora beticola* in Sugar Beets

**DOI:** 10.3390/jof11030211

**Published:** 2025-03-09

**Authors:** Erich-Christian Oerke, Ulrike Steiner

**Affiliations:** Institute of Crop Science and Resource Conservation—Plant Pathology, Rheinische Friedrich-Wilhelms-Universitaet Bonn, 53113 Bonn, Germany; u-steiner@uni-bonn.de

**Keywords:** asymptomatic colonization, hemibiotrophy, hyphal dimorphism, necrotrophy, toxin

## Abstract

The length of incubation period, i.e., the time between first contact of host and pathogen and the appearance of symptoms, varies among diseases and depends on environmental conditions. *Cercospora beticola* is the most important fungal pathogen in sugar beet production worldwide, as Cercospora leaf spot (CLS) reduces the leaf area contributing to yield formation. Using sugar beet cultivars differing in CLS resistance, a single infection period of *C. beticola* resulted in minor differences in the incubation period among host genotypes and among individual plants of cultivars, greater differences among leaves within plants, and substantial variation within individual leaves. Under greenhouse conditions not suitable for secondary infections, the first CLS lesions appeared 10 days after inoculation; however, the number of leaf spots and CLS severity further increased significantly for another 7 to 17 days. A geographic information system approach enabled the tracking of colony appearance and growth of all CLSs on inoculated leaves for up to 27 days. Asymptomatic colonization of leaves was associated with thick hyphae which switched to thin hyphae or melanization after lesion appearance. The lifestyle of *C. beticola*—intercellular tissue colonization, triggering of necrotic host reaction—is discussed considering the experimental results as well as literature resources.

## 1. Introduction

*Cercospora beticola* Sacc. (1879) is responsible for high yield losses in sugar beet production worldwide because it causes the formation of necrotic leaf spots leading to reduced recoverable sucrose [1]. The fungus is not known to produce a sexual stage, but genetic analysis confirmed membership within the Mycosphaerellaceae, order Capnodiales, class Dothideomycetes, phylum Ascomycota [2,3]. The fungus may be cultivated on artificial media, but survives in the field as desiccation-resistant pseudostromata within substomatal cavities of sugar beet debris on and in the soil only for a period of time of 2 years [4]. These authors named *C. beticola* a hemibiotroph, whereas Weiland and Koch [5] had classified it earlier as necrotroph. As the pathogen has a narrow range of host species—sugar beet, other cultivated and wild Beta species, and species of the genera *Malva*, *Spinacia*, *Limonium*, *Chrysanthemum*, *Lactuca*, *Carthamus tinctorius*, *Rumex obtusifolius*, and *Apium* [4,6]—it can be successfully managed by crop rotation. *Cercospora* leaf spot (CLS) is favoured by high temperatures and spells of high relative humidity, and the formation of dew during night-time may be sufficient for successful infection.

Germinating acicular, multi-septate conidia (2–4 × 40–200 μm) form thin (width 1.3 to 2.1 µm, average 1.7 µm) germ tubes that penetrate leaf tissue directly or via appressoria through stomata and spread intercellularly with no visible leaf symptoms [5,7,8,9]. The switch from this lifestyle to necrotrophy is reported to be related to the production of toxins and degradative enzymes which cause the necrotizing of infected tissue [9]. Symptoms appear as circular spots 3–5 mm in size and tan to grey in colour, often encircled by a tan–brown to red–purple border [10]. Tiny, slightly chlorotic depressions may develop as early as 5 to 6 days after inoculation (d p.i.) and enlarge to 1 mm in diameter by 10 to 13 d p.i. [9,11]. Substomatal pseudostromata within the lesions produce melanized conidiophores with hyaline conidia, which may be produced as early as 7 days after infection and 1 to 3 d after lesion appearance, respectively, under favourable conditions [12]. Conidiophores emerge only through stomata, and conidia are typically produced from primary infections within 7 to 21 days, depending on temperature, light, leaf age, and host resistance [13].

The epidemiology of the polycyclic CLS—the life cycle of *C. beticola*, optimal conditions for infection and spread, source of inoculum, and spread—is well understood [14,15,16,17]. Host plant resistance reduced the length of conidia, speed of germination, and spore yield, but had no effect on the latent period [14]. *C. beticola* may also be transmitted by seed material. The pathogen resides in the pericarp of sugar beet fruit rather than the true seed. Pathogen DNA could be detected in xylem sap, suggesting the vascular system is used to systemically colonize the host [18].

The incubation period (IP), i.e., the period of time between penetration and first appearance of disease symptoms on the host, is one component of partial resistance, which may also influence spore germination, penetration into the host tissue (summarized as infection frequency), colonization of the host tissue (=lesion size), the duration of latent and infectious periods, and sporulation [19,20]. In contrast to IP referring to the disease symptom, the latent period (LP) is an epidemiological parameter estimating the time between infection and the occurrence of new pathogen propagules (=generation period, infection cycle), which is highly relevant for disease forecast modelling. The latent period is often longer than IP, though in some cases it is equal [21]. For hemibiotroph pathogens, IP also characterizes the length of the initial biotroph stage, which is considered to be without visible symptoms, i.e., asymptomatic infection. In CLS disease, sporulation (i.e., the end of the latent period) depends on the availability of free water, and is induced by rainfall or high relative humidity [13]. The incubation period, therefore, is more suitable than the latent period to characterize the transition from symptomless leaf colonization to necrotrophy.

The incubation period of diseases varies significantly among host–pathogen interactions. It may vary between few days for necrotrophs like *Botrytis cinerea* and *Fusarium* species causing *Fusarium* head blight of cereals, 5–7 days for biotrophs like *Blumeria graminis* on barley and wheat, and *Plasmopara viticola* on grapevine, and 21–28 d for *Zymoseptoria tritici* causing *Septoria tritici* blotch on wheat. In all of these cases, IP variability depends on differences in host plant resistance and ambient temperature, and ranges from 1 to 3–7 days. In general, the time for symptom development decreases as temperature and disease susceptibility increases. In a meta-analysis on the relationship between trophic lifestyle of pathogens and the length of incubation and latent period, IPs (and LPs) of necrotrophs were shortest (<100 degree-days), biotrophs intermediate (100 to 200 DD), and hemibiotrophs had the longest periods (>200 DD): For *C. beticola*, the latent period was about 250 degree-days (DD) [21,22].

For *Cercospora* leaf spot, IP was reported to range from 11 to 13 days [23]. In the study by Rossi et al. [24], IP on a susceptible cultivar ranged between 4 and 9 days, depending on temperature. Afterwards, spots continued appearing on other leaves (!) over a 2–4 week period. Resistance delayed lesion appearance by a maximum of 12 days compared to the susceptible cultivar. Since leaf spots did not appear on leaves simultaneously, the dynamics of IP over time were analyzed by calculating the number of affected leaves on each day after inoculation. The IP_50_, i.e., the degree-day cumulation when 50% of leaves pass through IP, was 210 °C and 309 °C for the susceptible and the most resistant cultivar, respectively. Within-cultivar variability was an important source of variation; it was attributed to both ‘between plants’ (=genetic heterogeneity of cultivars) and ‘between leaves within plants’ (=differences in the age of the leaves of a plant) variability, accounting for 31% and 56% of total variability, respectively. In other reports, the number and size of CLS lesions were assessed once at the end of experiments.

In preliminary experiments with a single inoculation under controlled conditions, the number of leaf spots per sugar beet leaf significantly increased after the appearance of the first symptom(s) for more than 7 days. To our knowledge, within-leaf variability of the incubation period has been reported neither for CLS nor for other diseases, although this phenomenon is interesting, especially for the hemibiotroph pathogen *C. beticola*. The succession of the asymptomatic (biotroph) phase followed by a fine-tuned transition to necrotrophy characterizes *C. beticola* as hemibiotroph [4]. The two growth phases of hyphae within host tissue most probably require different effector repertoires. Effectors of *C. beticola* include toxins and proteinaceous effectors like a chitinase-binding protein [4], and *CbAve1* acting as a virulence factor [25]. Secretion of *C. beticola* necrosis-inducing protein 1 is able to induce necrosis within 48 h [26]. CbNIP1 was most active in complete darkness, as exposure of CbNIP1-infiltrated leaves with a 12 hr light–dark cycle led initially to chlorosis formation that gradually turned necrotic over time.

Cercosporin, a light-activated, nonhost-specific toxin produced by most *Cercospora* species, is reported as a virulence factor for *C. beticola* and other *Cercospora* species [4,27,28]. Application of cercosporin onto leaves resulted in lesion formation within 5 days, with lesions appearing similar to 10- to 13-day-old lesions resulting from *C. beticola* infection [29]. However, cercosporin-induced lesions lacked a well-defined boundary zone and general wall thickening associated with *C. beticola* infections. Cercosporin stimulates the generation of reactive oxygen species (ROS), and when ROS levels are high, cell structure collapses due to membrane lipid peroxidation and electrolyte leakage [27]. Mutants of the cercosporin biosynthesis gene *CbCTB2* lacked pigmentation, and failed to produce cercosporin and to colonize sugar beet leaves [30]. It was discussed to be involved not only in the necrotic stage during later stages of pathogenesis, but also to play a role in early pathogenesis, probably by suppressing host resistance [30]. Beticolins are another group of non-host-specific toxins; twenty beticolin compounds of *C. beticola* have been identified, but the biosynthetic pathways and toxicity are still unclear [4]. In addition to the toxins, virulence factors of *C. beticola* may include cellulases, pectinases, and melanin [31,32].

Observation of *C. beticola* structures within sugar beet leaf tissues has been limited by the efficacy of staining techniques [33]. Conventional fungal staining techniques are not effective because the dyes do not penetrate into the leaf tissue. Intermediate steps of staining protocols may include heat treatments that may cause tissue disruption and artefacts. Bhuiyan et al. [33] used Alexa Fluor-488-WGA staining and confocal microscopy for studying the initial 120 h p.i. of *C. beticola* on a susceptible and a resistant host cultivar; however, the images presented are inconclusive on whether the fungal biomass in the leaf tissue was included.

The aim of this study was the characterization of the early, latent stage of *C. beticola* by investigating the variation of the incubation period of typical *Cercospora* leaf spots on the leaf level, as this is equivalent to the duration of biotroph/asymptomatic infection. The increase in the number of CLS lesions and their spatial patterns were assessed for individual leaves of sugar beet cultivars varying in CLS resistance by using a geographic information system (GIS) approach, which allows for the observation of all lesions of a leaf over time individually. The Global Positioning System (GPS) has been used in the field for sampling and *C. beticola* genotyping on the plant scale [34]. Here, GIS was used on the leaf scale for longitudinal imaging and monitoring of individual leaf spots retrospectively. Also, the duration of active leaf spot growth was assessed. Microscopic investigations of leaf spots and tissue outside of leaf spots immediately after their appearance and during the subsequent necrotroph stage should yield new information on growth and nutrient uptake of the hemibiotrophic pathogen. It was assumed that infection structures formed during asymptomatic tissue colonization still exist immediately after symptom appearance. They may be modified and accompanied by new pathogen structures typical for the necrotroph stage. As molecular labelling may interfere with *C. beticola* fitness, non-stained specimens had to be observed in many cases.

## 2. Materials and Methods

### 2.1. Plants

Several cultivars of sugar beet (*Beta vulgaris* L. subsp. *vulgaris* var. *altissima* Döll) were used in greenhouse experiments in order to consider host genotypes differing in susceptibility (and resistance mechanisms) to CLS: cv. Brix (Strube D&S GmbH, Söllingen, Germany) with CLS susceptibility score 5, according to [35]; cv. Carsta (KWS Saat SE, Einbeck, Germany; score 2); cv. Debora (KWS, score 5); cv. Elaina (KWS, score 2), cv. Emilia (KWS, score 5); cv. Pauletta (KWS, score 4). Plants were grown from seeds in commercial potting substrate (Einheitserde, Klasmann-Deilmann GmbH, Geeste, Germany) in a greenhouse at 23/20 °C (day/night), 50–60% relative humidity (RH) and a photo-period of 16 h (>300 µmol m^−2^s^−1^) per day. Plants were watered as necessary and fertilized weekly with 100 mL of a 0.2% solution of Poly Crescal (Aglukon GmbH, Düsseldorf, Germany). They were used for experiments when they had reached growth stage BBCH 14 [36].

### 2.2. Pathogen Inoculum and Inoculation

CLS-diseased sugar beet leaves were sampled in a field near Bonn, Germany, and inoculum was propagated on susceptible host genotypes under greenhouse conditions. Dried diseased leaves were stored in the dark at room temperature. Inoculum of *Cercospora beticola* Sacc. was harvested from wetted leaves incubated at 100% RH for 48 h. Conidia were washed off with a 0.01% solution of polysorbate 20 (Tween 20, Sigma-Aldrich, Munich, Germany), and a spore suspension with 2–4 × 10^4^ conidia mL^–1^ was sprayed onto sugar beet leaves. In experiments on the effect of the length of leaf wetness period, plants were incubated at 100% RH for 1, 2, 3, 4, and 7 days after inoculation (d p.i.). After incubation at 100% relative humidity (RH) and 25 °C/20 °C day/night temperature for 48 h, plants were put back to 60 ± 10% RH for up to 4 weeks in order to prevent pathogen sporulation and new infections. Leaf wetting of inoculated leaves was strictly avoided by watering the plants from below.

### 2.3. Disease Assessment

In all experiments, the appearance and development of CLS lesions was investigated on leaves attached to the potted plants. The number of CLS lesions was assessed visually on a daily scheme considering lesions with a (light) brown–grey centre and a well-defined border to green leaf tissue only. Unspecific yellow spots and tissue indentions occurring previously were not considered for quantitative CLS assessment. The diseased leaf area was estimated as percentage leaf area covered using a scale with 0, 1, 3, 5, 10, 20, …, 100% diseased leaf area. The area of individual CLS lesions was measured after imaging of diseased leaves with a binocular (Leica MZ 16 F, Leica Biosystems, Wetzlar, Germany) and using the DISKUS software (vers. 4.6, Hilgers Technisches Büro, Königswinter, Germany) for manual encircling of lesions.

For digital image-based disease assessment, diseased plants were transferred daily into a darkened room with a white LED light source in order to take images of leaves with CLS lesions with a Nikon D50 camera (Nikon Deutschland, Düsseldorf, Germany). Leaves were fixed between two grids (mesh size 20 × 20 mm) made from black fibres to support the leaves from below and to smooth them from above; a blue background was placed below the leaf. The images were analyzed using the ASSESS 2.0 image analysis software for plant disease quantification (Assess 2.0; L. Lamari, American Phytopathological Society, St. Paul, MN, USA) as described by [37] and in the software’s user manual. In addition to leaf area and leaf area with symptoms, the number of CLS lesions per leaf and the area of individual CLS lesions were computed. The data were analyzed after transfer to Excel (Microsoft Deutschland, Munich, Germany).

### 2.4. Geographic Information System for Longitudinal Assessment of CLS

For recording a longitudinal series of images of infected sugar beet plants, individual leaves of cultivars Carsta, Debora, Emilia, and Pauletta were fixed between two grids of black fibres. Images covering the total leaf area were taken from the same leaves after the appearance of first CLS lesions and the next 7, 11, and 17 days for cvs. Carsta and Debora, Pauletta, and Emilia, respectively. Differences among cultivars were due to the start of senescence and tissue yellowing. The series of 5 (Carsta), 6 (Debora, Pauletta), and 10 (Emilia) images were processed and analyzed using the software ArcGIS Desktop 10.8.2 (ESRI Deutschland GmbH, Kranzberg, Germany). Using the first image of the series as template, the following images were spatially referenced using the geo-referencing tool. The outline of the leaf and the CLS lesions were digitalized manually. For identification of individual lesions within the longitudinal image series, a number was assigned to each CLS lesion of the leaf. Information on the spatial position and the area of the objects were analyzed after data transfer to Excel.

### 2.5. Microscopic Investigations

All microscopic investigations were repeated at least two times. For each experiment, samples for microscopic investigations were taken from similar leaves from at least 4 sugar beet leaves. Microscopic images are representative for sugar beet leaf tissue and the fungal structures indicated. Leaf discs for microscopy were sampled in experiments different from those using a GIS approach.

Leaf discs were excised from sugar beet leaves by using a cork borer (Ø 11 mm). They were cleared in saturated chloral hydrate (Sigma-Aldrich, Darmstadt, Germany) for 3 to 5 days. For bright-field microscopy, cleared samples were stained with toluidine blue (0.005% toluidine blue in 0.01 M phosphate buffer, pH 6.0). For fluorescence microscopy of fungal structures and callose, leaf samples were rinsed with distilled water and stained with aniline blue (0.05% aniline blue in 0.067 M K_2_HPO_4_) according to [38]. For imaging of total preparations, samples were studied under the light microscopes DMRB and DM6000 B (Leica, Wetzlar, Germany), equipped with Nomarski interference contrast and epifluorescence, respectively. The aniline-blue-stained pathogen structures were visualized using filter cube A (excitation 340–380 nm, beam splitter 400 nm, stop filter LP 425 nm). Images were recorded and analyzed using the software Diskus (v. 4.60.1611; Technisches Buero Hilgers, Koenigswinter, Germany).

### 2.6. Statistical Analysis

All experiments were repeated at least two times. Experimental data were analyzed using the software SPSS (IBM SPSS Statistics 15, Armonk, NY, USA). All tests were conducted using a significance level α = 0.05. As data were not normally distributed, they were analyzed using the Kruskal–Wallis H test, sample means were tested for significant differences by applying pairwise comparisons respecting the Bonferroni correction. Box-and-whisker plots were used to demonstrate the variation within classes and class medians.

The variance-to-mean ratio (VMR) was used to characterize the spatial pattern of CLS lesions. Segmentation of the leaf area into areas of similar size by applying a grid adapted to the leaf’s size and shape to the digitalized image resulted in 20, 23, 25, and 27 grids (subareas) for cvs. Emilia, Carsta, Debora, and Pauletta, respectively. The number of CLS lesions per grid at the time of first appearance and 7 (17) days after appearance were used for VMR calculation. The spatial pattern was considered to be homogenous, random, and clustered for VMR values <0.8, 0.8 to 1.2, and >1.2, respectively.

## 3. Results

### 3.1. Variation in the Length of Incubation Period of CLS Lesions

**Figure 1:**
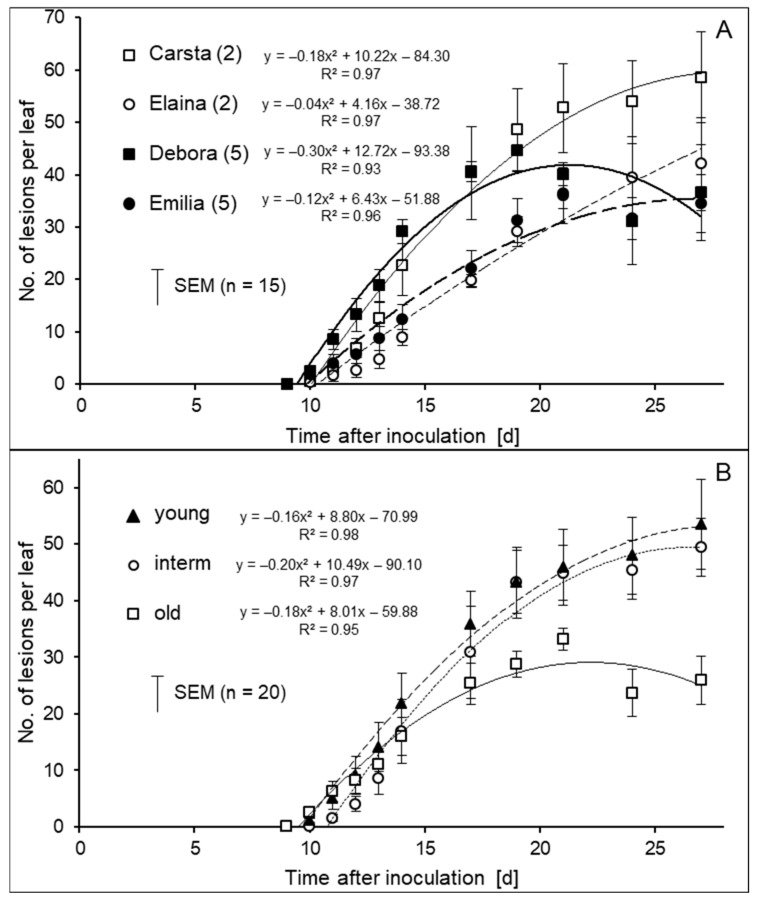
Increase in the number of CLS lesions of sugar beet leaves depending on the disease susceptibility of cultivars (**A**) and the ontogenetic stage of leaves (**B**). Two cultivars with low (score 2) and moderate (score 5) CLS susceptibility, respectively, and three leaf ages (young; level 2 from top); intermediate, level 4 from trop; old, level 6 from top) were assessed for typical CLS lesions over a period of 8 to 27 d p.i. (4 cvs. × 3 leaf levels × 5 replicates).

Sugar beet plants grown for 48 h under optimum infection conditions—100% RH, day/night cycle—exhibited the first non-specific symptoms (tiny yellow spots) 7 to 9 days after inoculation, which developed into typical CLS lesions (light brown to grey leaf spots encircled by a reddish-brown margin within two days). These typical CLS lesions were used for the assessment of the length of the incubation period. Continuous appearance of additional CLS lesions within a period of another 17 days demonstrated a significant variability in the lesions’ incubation period (Figure 1). The effect was influenced by the host genotype and the ontogenetic status of leaves; however, the increase was most pronounced within the first 7 to 10 days, and then levelled off in all cases. For the first and last symptom(s), the length of incubation period varied by factor 3. On susceptible genotypes, CLS lesions had a minimum incubation period 1 d shorter than on partially resistant genotypes, which showed the strongest increase in the number of CLS lesions in the second half of the observation period (Figure 1A). Older leaves were more susceptible than young leaves—first symptoms appeared 1 d earlier—but the number of CLS lesions remained lower than on upper leaf levels where CLS lesions reached a higher asymptotic level only at the end of the observation period (Figure 1B).

### 3.2. Development of Lesion Growth Depending on Age and Size

**Figure 2:**
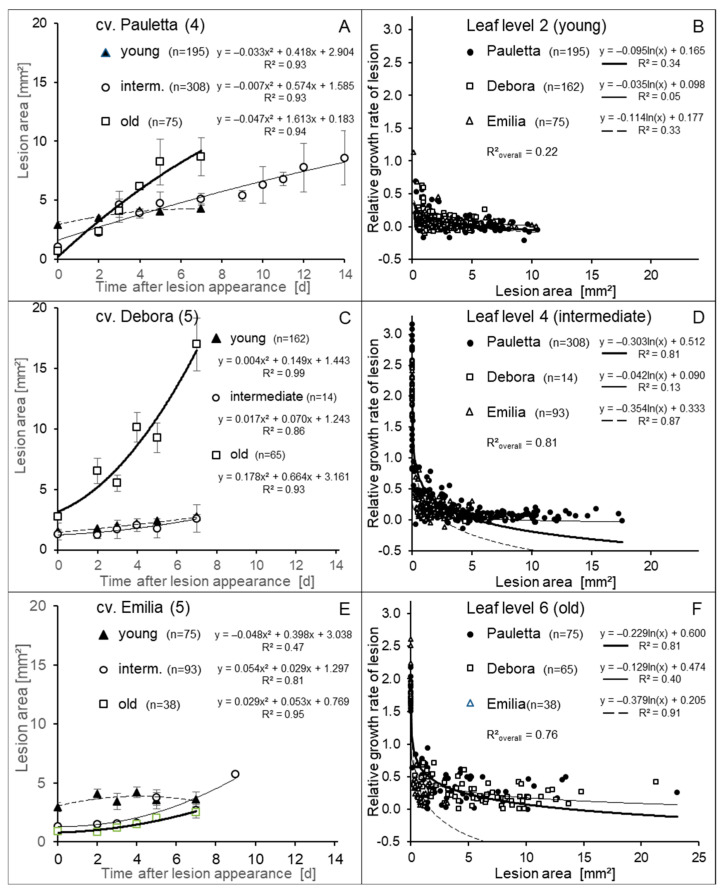
Growth of CLS lesions after the time of first appearance depending on sugar beet cultivar and leaf age (**A**,**C**,**E**) and relationship between lesion size and relative growth rate (RGR; for two days) of lesions depending on leaf age (**B**,**D**,**F**).

As coalescence of CLS lesions was described to substantially contribute to leaf damage, the growth of individual CLS lesions was assessed after their first appearance on the three leaf levels of three sugar beet cultivars. A large-scale expansion of lesions to cover large leaf portions would indicate to extensive necrotrophic growth of the hemibiotroph pathogen. Within a period of 7 to 14 days after appearance, the lesion area increased from the initial 2 to 3 mm^2^ to a maximum of 10 mm^2^ (Ø 3.6 mm), 17.5 mm^2^ (Ø 4.7 mm), and 6.5 mm^2^ (Ø 2.9 mm) on cvs. Pauletta, Debora, and Emilia, respectively. Lesion growth was strongest on old leaves and lesser on young leaves (Figure 2A,C,E). The relative growth rate of CLS lesions revealed a strong negative effect of lesion size on further growth for all ontogenetic leaf stages (Figure 2B,D,F). This effect was most pronounced for young leaves of cvs. Pauletta and Emilia. The lower coefficient of determination (R^2^) for cv. Debora indicated a deviation for this sugar beet genotype.

### 3.3. Effect of the Length of Optimal Infection Conditions on CLS Intensity and Epicuticular Development of C. beticola

**Figure 3:**
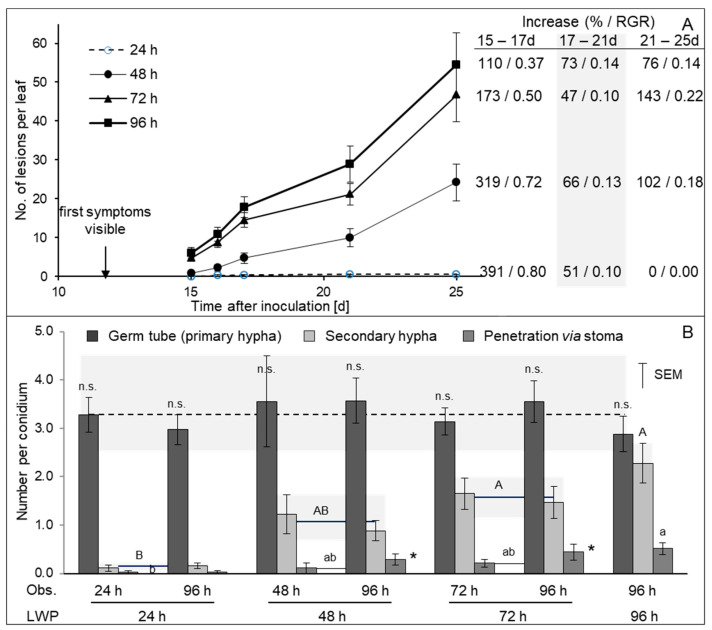
Effect of length of initial leaf wetness period on the number of CLS lesions 15 to 25 d p.i. and increase in the number of lesions over time expressed as % increase and relative growth rate (RGR), respectively (cv. Brix, n = 33; (**A**)). Growth of *C. beticola* on the leaf surface; formation of primary and secondary germ tubes and successful penetration via stomata 24, 48, 72, and 96 h p.i. (**B**). Grey background visualizes the range of parameter variability (n = 30; Obs., Time of leaf sampling; LWP, leaf wetness period). Different letters represent statistical significant (*p* ≤0.05) differences between average values for 24, 48, 72, and 96 h leaf wetness duration (upper case letters for secondary hyphae, lowercase letters for penetration via stoma); asterisk indicates significant difference (*p* ≤ 0.05) between observations made directly after leaf wetness and 96 h, respectively.

In addition to variations in genotypic and ontogenetic disease resistance of sugar beet, vitality differences in the conidia population used as inoculum may cause differences in IP length of individual lesions on leaves of similar susceptibility. Variation in the length of the initial leaf wetness period demonstrated that the number of CLS lesions per leaf increased with both the length of optimal infection conditions and the time after the appearance of first symptoms (Figure 3A). Leaf wetness for 24 h resulted in minimum CLS intensity, which increased from 0.03 (1 out of 33 leaves with a symptom) to 0.56 (9 leaves with symptoms) within a period of 6 days. Leaf wetness for 48, 72, and 96 h, respectively, significantly increased the number of symptoms 15 d p.i., whereas the relative growth rate of the lesion number was similar for all leaf wetness periods. A leaf wetness period of 7 days resulted in even more CLS lesions; however, caused also direct plant damage because of the susceptibility of sugar beet to flooding conditions.

Microscopic investigations of the epicuticular development of *C. beticola* demonstrated that conidia germination was completed within 24 h (Figure 3B). On average, 3.2 germ tubes (primary hyphae) were formed from individual cells of the multi-septate conidia, irrespective of the length of leaf wetness. The number of secondary hyphae significantly increased with leaf wetness length, and was 14 times higher for 96 h than for 24 h. Similarly, the number of leaf penetrations via stoma increased from 0.03 to 0.52 per conidium. Data for 48 and 72 h leaf wetness were between the extremes. Comparison of observations directly after the end of these leaf wetness periods and 96 h demonstrated a significant (*p* < 0.05) increase in the number of stomata penetrations in the time between the end of leaf wetness 48 and 72 h and 96 h p.i.

### 3.4. Appearance and Growth of CLS Lesions on Individual Sugar Beet Leaves

Previous results pointed to the necessity to observe individual disease symptoms in longitudinal studies in order to learn more on the large variation in the incubation period of CLS lesions, which is equivalent to the asymptomatic phase of individuals of the hemibiotroph *C. beticola*. Techniques from geographic information systems were applied to images of infected leaves in order to assess the spatial and temporal patterns of appearance and growth of all CLS lesions on individual leaves of four sugar beet cultivars (Figures S1 and S2).

**Figure 4:**
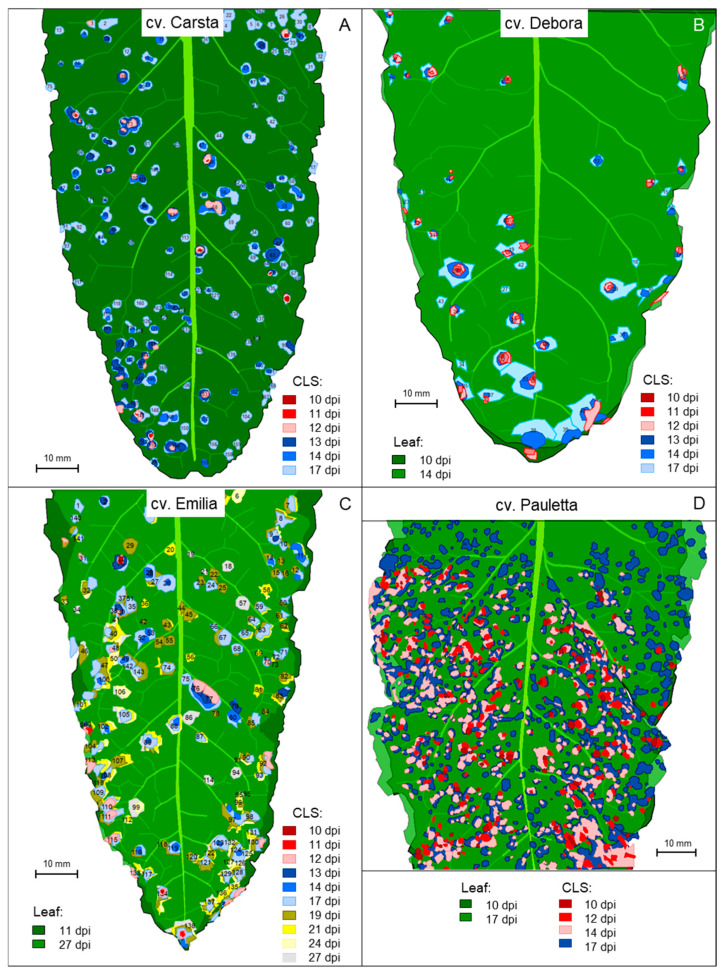
Appearance and development of individual CLS lesions on leaves of four sugar beet cultivars; (**A**) cv. Carsta in the period 10 to 17 d p.i.; (**B**) cv. Debora in the period 10 to 17 d p.i.; (**C**) cv. Emilia in the period 10 to 27 d p.i.; (**D**) cv. Pauletta in the period 10 to 17 d p.i. The colour code represents the time of lesion appearance; the size of lesions represents lesion growth in time. As shape and area of leaves were affected by disease and time, two leaf outlines are given for the leaf of cvs. Debora, Emilia, and Pauletta, respectively. Numbers within lesions indicate the numbering of lesions during data processing.

**Figure 5:**
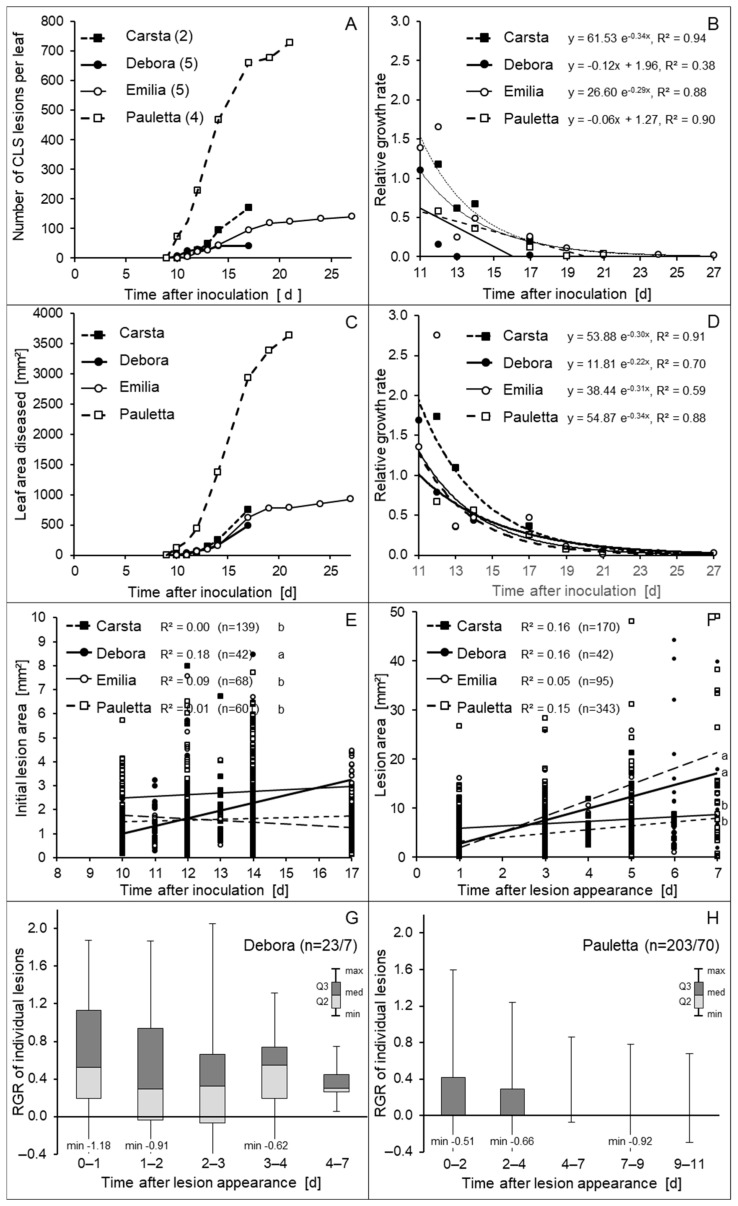
Development of CLS lesions on leaves of four sugar beet cultivars in time. Increase in the number of CLS lesions per leaf in the period 9 to 27 d p.i. (**A**); relative growth rate (RGR) of lesion number over time (**B**); increase in the total leaf area diseased in the period 9 to 27 d p.i. (**C**); relative growth rate (RGR) of diseased leaf area (**D**); effect of the length of incubation period on the initial area of CLS lesions (**E**); growth of CLS lesions within the period of 7 days after the time of appearance (**F**); box-and-whisker plots of the relative growth rate of individual CLS lesions on cv. Debora (**G**) and Pauletta (**H**) in response to the time after lesion appearance. Assessment of lesions appearing 10 and 11 d p.i. (cv. Debora) and 10 and 12 d p.i. (cv. Pauletta), respectively. Lower case letters indicate significant differences (*p* ≤ 0.05) among cultivars (**E**,**F**).

On leaves of cvs. Debora, Emilia, and Pauletta, first symptoms—8, 1, and 73, respectively—appeared after 10 days, whereas the less susceptible cv. Carsta had the first CLS lesion at 11 d p.i. (Figure 4, Table 1). First symptoms preferentially appeared on intercostal areas, and not on the leaf margin, which often became symptomatic in later stages. These colonized leaf margin areas dried out very soon, and resulted in shrinking and undulation of leaves. Within 7 (6 for Carsta) days after appearance (d p.a.) of the first symptom(s), the number of CLS lesions for cvs. Carsta, Debora, Emilia, and Pauletta increased by 162, 35, 94, and 589, respectively. Observations for cvs. Pauletta and Emilia for 11 and 17 d p.a. proved an even longer IP for 57 and 46 lesions, respectively, i.e., the number of lesions increased by 14.3 and 4.6 lesions per day as compared to 84.1 and 13.4 lesions per day within the first 7 d p.a. (Figure 5A). The relative growth rate of the number of CLS lesions and the total leaf area diseased decreased exponentially with time (Figure 5B,D).

The initial size of CLS lesions at the day of appearance increased significantly only for cv. Debora (r = 0.42) the leaf of which exhibited indications of senescence during the time of observations (Figure 5E). In contrast, the correlation between the time after inoculation and lesion area was negligible or low (cv. Emilia) for the other sugar beet genotypes. Nevertheless, the area of individual lesions expanded with the time after lesion appearance on all cultivars with cv. Emilia allowing the lowest lesion growth (Figure 5F). RGR of individual CLS lesions decreased with the time after appearance, and the median RGR of lesions on cvs. Carsta, Emilia, and Pauletta was 0 as early as 3 to 5 d p.a. Although RGR decreased with time as well on the old leaf of cv. Debora, CLS lesions still expanded at 7 d p.a. (Figure 5G,H and Figure S3). The box-and-whisker plots demonstrated that a considerable number of CLS lesions did not expand at all; negative RGR values for individual lesions resulted from the shrinkage of leaves due to the disease and senescence. Mean growth duration of CLS lesions until 17 d p.i. was 5.8, 4.9, 2.5, and 2.8 d for cvs. Carsta, Debora, Emilia, and Pauletta, respectively (Table 1).

**Figure 6:**
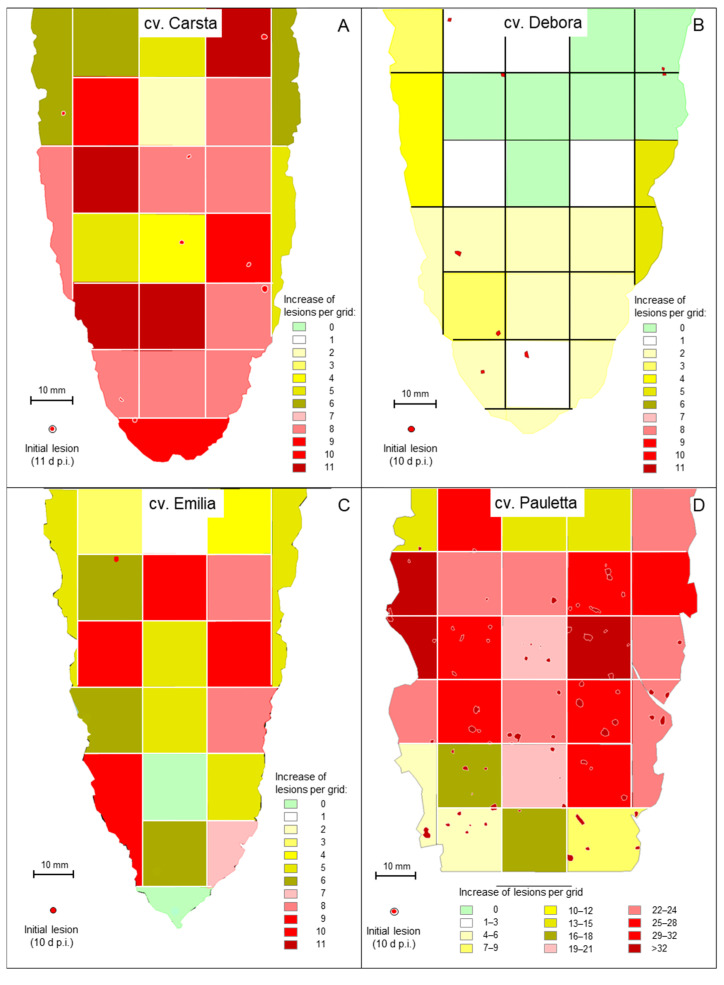
Spatial patterns of CLS on individual leaves of four sugar beet varieties; (**A**) cv. Carsta; (**B**) cv. Debora; (**C**) cv. Emilia; (**D**) cv. Pauletta. First lesions (red dots) 10 d p.i. (cv. Carsta 11 d p.i.) and spatial variability in the increase of CLS lesions within the period 10 to 17 d p.i. Colour coding represents the intensity of formation of additional CLS lesions per grid within a period of 7 days.

Spatial patterns of first CLS lesions and symptoms 17 d p.i. differed among cultivars and with time (Table 2). The initial pattern of CLS lesions at the time of first appearance was homogenous and random, except for cv. Pauletta exhibiting a clustered lesion pattern at high CLS incidence. At 17 d p.i., the lesion patterns of all cultivars had shifted to randomness and aggregations. This effect resulted from the random or aggregated increase in CLS lesions with VMR values ranging from 0.75 for cv. Carsta to 2.84 for cv. Pauletta. The additional CLS lesions appeared with no preference whether the area had early lesions or not—for the four leaves, the mean increase for grids with and without primary lesions was 9.2 and 8.2, respectively. The visualization of the increase of CLS lesions within 7 days—6 days for cv. Carsta—highlighted spatial differences in the increase within leaves (cultivars) depending on genotypic CLS susceptibility and the fact that the level of increase was independent from the presence of initial CLS lesions (Figure 6).

### 3.5. Spatial and Temporal Patterns of C. beticola Structures in Sugar Beet Leaves

**Figure 7:**
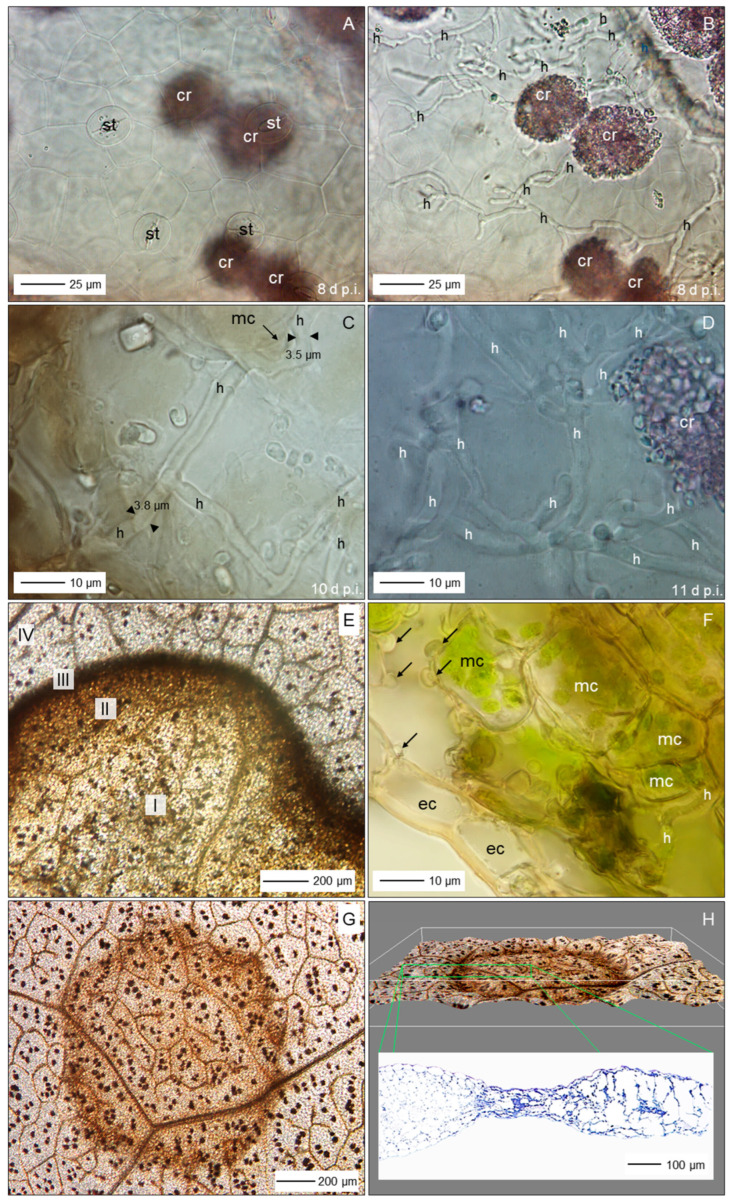
Characteristics of sugar beet leaves infected by *C. beticola*. Surface of cleared leaf tissue without disease symptoms, 8 d p.i.; st, stoma; cr, crystal inclusions (**A**); same site with focus a little bit lower demonstrating intensive colonization by *C. beticola* hyphae (h; (**B**)); close-up of non-melanized intercellular hyphae with a diameter about double that of epicuticular germ tubes (**C**,**D**); subareas of sugar beet leaf tissue with a CLS lesion; I, lesion centre; II, intermediate; III, (brown) margin; IV, green leaf area outside the lesion (**E**); cross section of sugar beet tissue at the margin of a CLS lesion 14 d p.i.; epidermal cells (ec) and mesophyll cells (mc; with chloroplasts) form globular protuberances (**F**); top view (**G**) and side view (**H**) of a CLS lesion visualizing tissue breakdown of the margin and the less pronounced damage of the lesion centre. Bright-field microscopy after staining with toluidine blue ((**A**–**D**,**H**), lower part).

Microscopic investigations on indented leaf anomalies 8 d p.i. demonstrated an intact epidermal layer and crystal inclusions of sugar beet tissue the mesophyll of which was intensively colonized by *C. beticola* hyphae with a diameter of >3.5 µm (Figure 7A–D). Their growth was not affected by calcium oxalate crystal deposition in vacuoles. Hand sections of early CLS lesions revealed globular protuberances of epidermal cells and mesophyll cells with intact chloroplasts outside the (reddish-)brown margin of lesions which showed three different subareas well-defined from the green tissue (Figure 7E,F). In 3D illustrations of nadir images of CLS lesions and cross sections of lesions, the leaf tissue of the brown margin proved to be collapsed and shrivelled whereas the lesion centre had almost the diameter of the healthy tissue outside of the margin (Figure 7G,H). The transition from necrotic to unaffected tissue at lesion margins was characterized by fortified host cell walls as visualized by autofluorescence (Figure S4).

**Figure 8:**
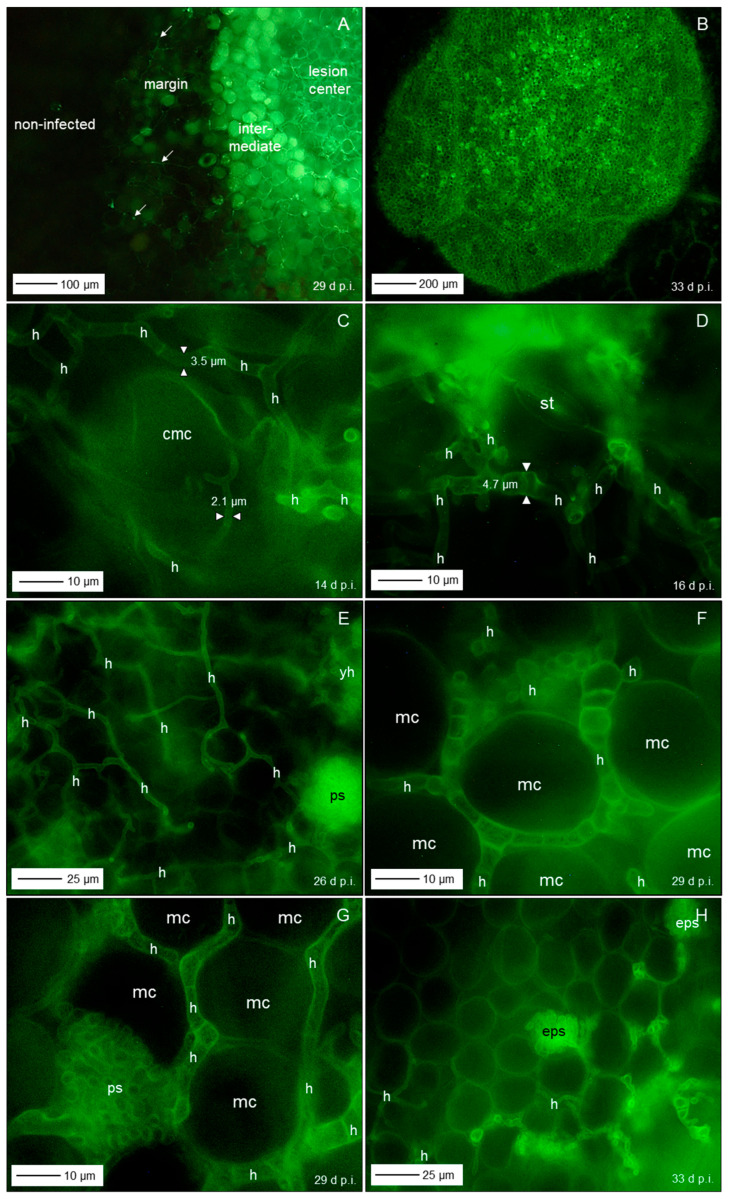
Colonization of leaf tissue by *C. beticola*. Subareas of CLS lesion and neighbouring tissue; arrows point to hyphal structure of margin and beyond (**A**); total lesion with cellular organization of leaf tissue, 33 d p.i. (**B**); hyphae with diameter ranging from 3 to 5 µm intercellularly colonized the lesion centre (**C**,**D**,**F**) eventually forming early pseudostromata above the mesophyll tissue (**E**,**G**,**H**). Epifluorescence microscopy after staining with aniline blue. cmc, collapsed mesophyll cell; eps, early pseudostroma; h, hypha; mc, mesophyll cell; st, stoma; yh, yeast-like hypha.

Fluorescence of aniline-blue-stained cell wall carbohydrates revealed also the three lesion subareas and the surrounding healthy tissue with intercellular fungal hyphae, especially in the lesion centre; in some cases, hyphae were also detected in the margin tissue and beyond (Figure 8A,B). Higher magnification revealed intercellularly growing hyphae with a diameter >3 µm and some narrow hyphae (Ø < 2.5 µm) colonizing substomatal cavities (Figure 8C,D). The diameter of intercellular hyphae (2 to 6 µm) differed from the very narrow (Ø < 2 µm) epiphytic primary (germ tubes) and secondary hyphae (Figure S5). The intensity of intercellular colonization of sugar beet leaf tissue by *C. beticola* increased during pathogenesis and resulted in the formation of pseudostromata located above the mesophyll layer which were characterized by the aggregation of small yeast-like cells. Surprisingly, in these advanced stages of colonization, the cellular structure of the mesophyll was also hardly affected (Figure 8E–H).

**Figure 9:**
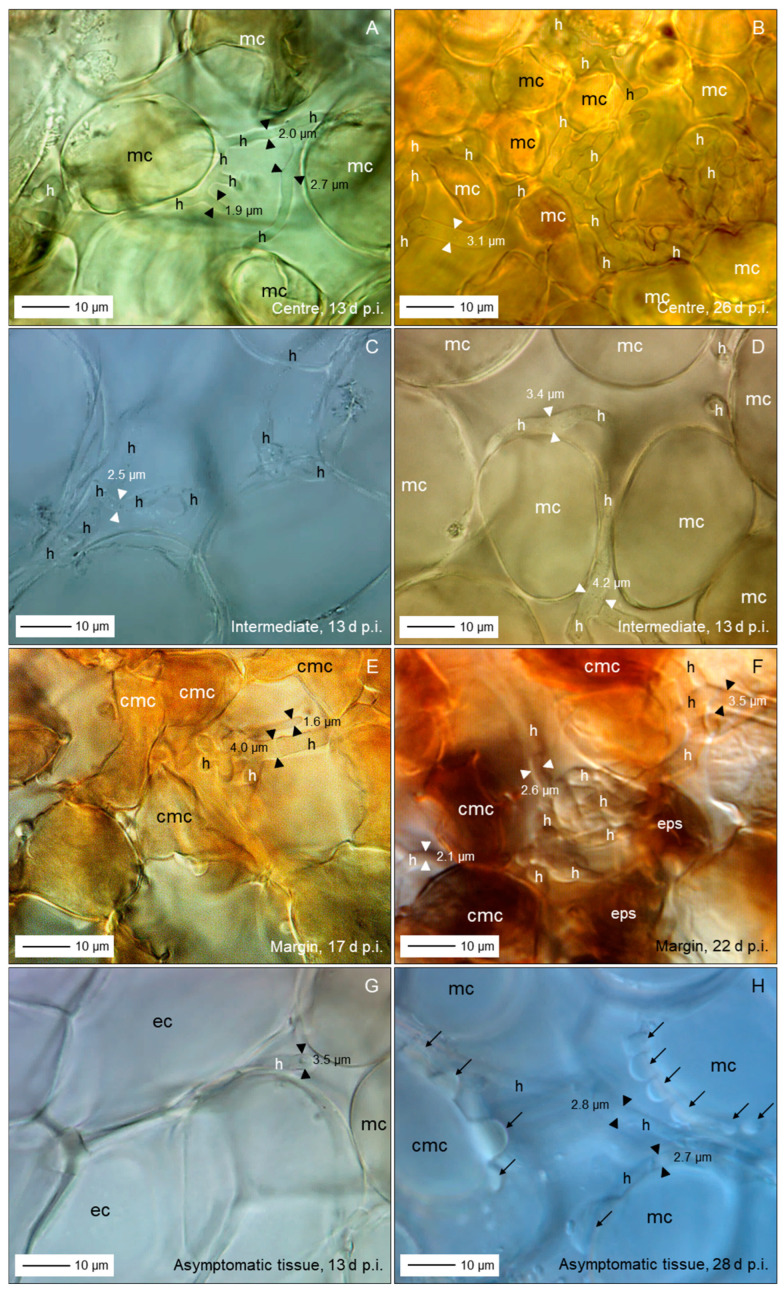
Differences in the tissue colonization of subareas of CLS lesions early after CLS lesion appearance (**A**,**C**,**D**,**E**,**G**) and in a later stage of pathogenesis (**B**,**F**,**H**). Intensity of intercellular colonization of leaf centre increased with time (**A**,**B**); intercellular hyphae in the intermediate region hardly damaged mesophyll cells (**C**,**D**); intercellular growth of hyphae among necrotic cells of the reddish-brown margin increased with time (**E**,**F**); leaf tissue outside the CLS lesion had minimal intercellular hyphae and hardly any cell damage (**G**,**H**). Bright-field microscopy after tissue clearing with chloral hydrate. cmc, collapsed mesophyll cell; ec, epidermal cell; eps, early pseudostroma; h, hypha; mc, mesophyll cell.

Bright-field microscopy of the subareas of CLS lesions and the adjacent green tissue in an early and advanced stage of pathogenesis—2 to 5 d and 10 to 16 d after lesion appearance, respectively—revealed a substantial increase of fungal biomass in CLS lesion centres and a smaller increase also in the margin tissue (Figure 9A,B,E,F). The rather wide diameter of *C. beticola* hyphae occasionally detected in the tissue surrounding the marginal ring indicated to a biotroph feature (Figure 9G,H). Tissue damage and cell death were obvious for the brown margin and the degree of tissue disintegration increased for lesion centres. Leaf cells outside CLS lesions often had globular protuberances into the intercellular space and the healthy-looking tissue included some collapsed mesophyll cells.

**Figure 10:**
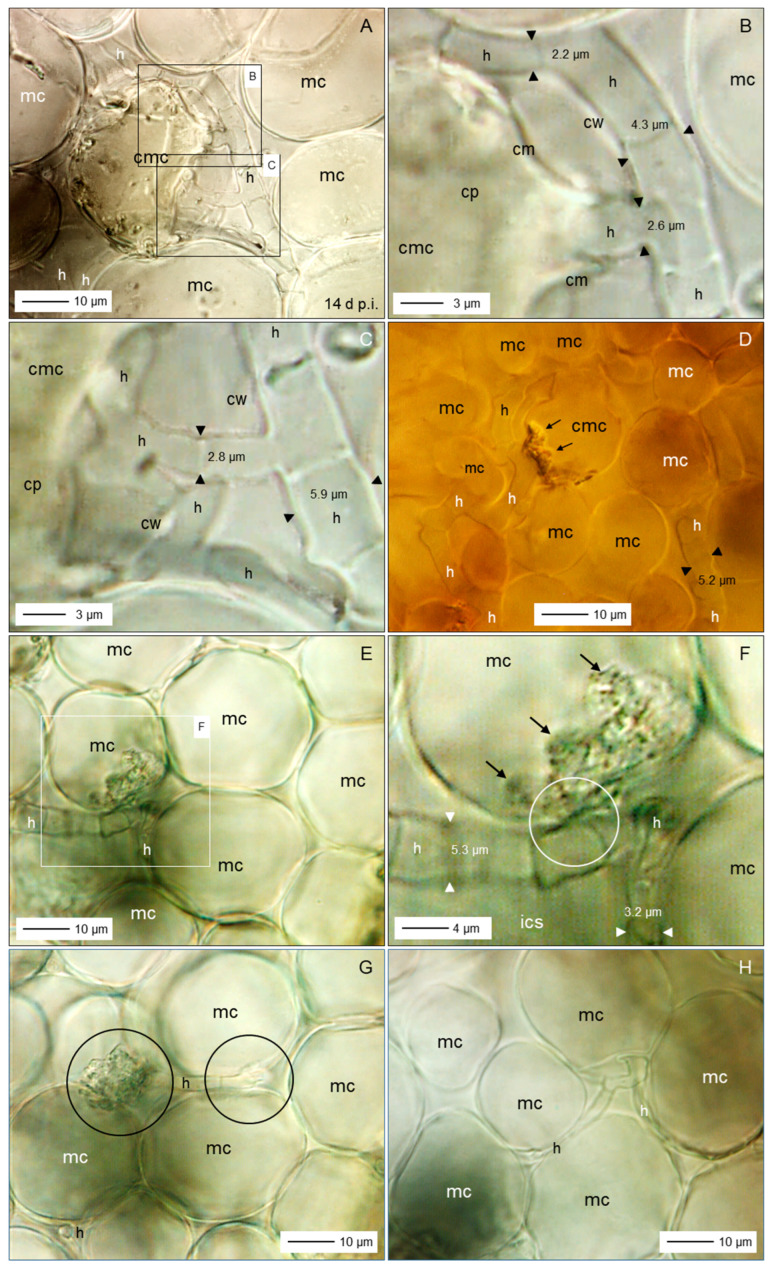
Details on intercellular growth of *C. beticola* hyphae in the centre of CLS lesions, 14 (**A**–**D**) and 17 (**E**–**H**) d p.i. Hyphae (h) with a diameter >5 µm grew between mesophyll cells that reacted to cell penetration by (thinner) hyphae with collapse of individual cells; neighbouring cells were not affected. cmc, collapsed mesophyll cell; cp, cytoplasm; cw, cell wall; mc, ics, intercellular space; mesophyll cell. Squares indicate to areas presented at higher magnification in the next image(s); circles and arrows highlight interesting areas and structures, respectively.

Short time after lesion appearance, intercellular development of *C. beticola* with hyphae 4 to 6 µm in diameter most often caused no visible effect on the mesophyll cells; nevertheless, hyphae that were considerably narrower (Ø < 3 µm) branching off primary intercellular hyphae penetrated into mesophyll, sometimes with two hyphae in a single host cell (Figure 10A–C). The penetration was associated with cell death which most probably was caused by penetration of the cytoplasm membrane. Death of plant cells in contact with wide hyphae was associated with a switch to growth with narrow hyphae (Figure 10D–F). In other cases, contact with plant cells was linked to growth with a narrower hypha, but not with cell death (Figure 10B). Cell reactions to narrow *C. beticola* hyphae varied within a short distance and in rare cases the mesophyll cell exhibited no disintegration of cytoplasm (Figure 10G,H).

**Figure 11:**
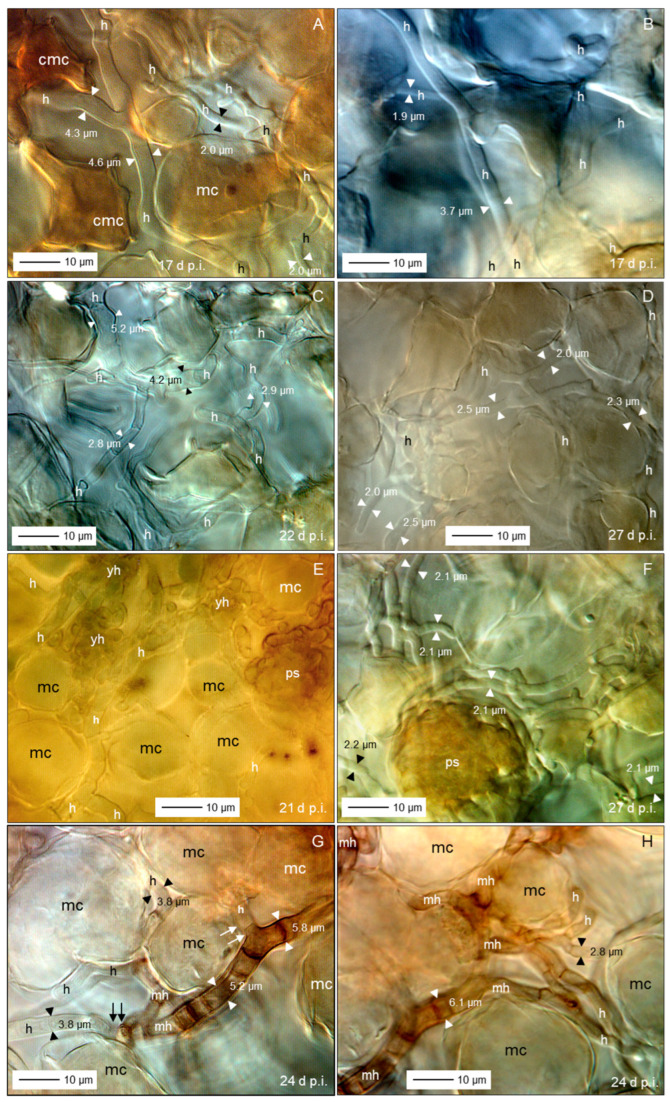
Variability of *C. beticola* hyphae growing in the centre of CLS lesions. In early lesions, hyphae with a diameter > 3.5 µm predominated (**A**,**B**). Hyphae formed after lesion appearance were narrower and became predominant in older lesions (**C**,**D**). The formation of early pseudostromata was associated with the formation of yeast-like shorter hyphae (yh), which became melanized in later stages of leaf colonization (**E**,**F**). Filamentous intercellular hyphae became melanized as well; they formed thinner, non-melanized hyphae which penetrated mesophyll cells (**G**,**H**). Bright-field microscopy after tissue clearing with chloral hydrate. Arrows indicate the transition from melanized to non-melanized hyphal cells.

Early after the appearance of typical CLS lesions, infected mesophyll tissue of the lesion centre was colonized predominantly by wide hyphae and hyphae with a diameter <2.5 µm in close vicinity were rare (Figure 11A,B). In more advanced stages, the portion of hyphae with a wide diameter decreased as new hyphae were narrow (Figure 11C,D). Pseudostromata as the base of sporulation under favourable environmental conditions were initiated by yeast-like hyphae formed in substomatal cavities above the mesophyll or between mesophyll and epidermal layer. At the time pseudostromata became melanized, wide *C. beticola* hyphae could be still observed, but were rare (Figure 11E,F). Melanization of narrow hyphae forming young pseudostromata in substomatal cavities could be observed as early as 17 d p.i. In contrast, intercellularly growing hyphae became melanized only at later stages of tissue colonization. Melanized wide hyphae formed narrow, melanized and non-melanized hyphae which penetrated mesophyll cells. Melanization of hyphal cells varied in hyphal strands in close vicinity; nevertheless, narrow, non-melanized hyphae were advancing from wide, melanized segments (Figure 11G,H).

## 4. Discussion

### 4.1. Length of Incubation Period

CLS lesions on sugar beet leaves had a rather long and highly variable incubation period (IP) of *C. beticola*, which completes its anamorph life cycle by the production of conidia in the centre of necrotic leaf spots if RH is close to 100%. Under moderate temperature conditions, IP of typical CLS lesions ranged from 10 to 11 d p.i.—for first symptoms, depending on partial resistance of the host genotype—to more than 25 d p.i. within individual leaves. Length of the asymptomatic phase of individual infections of *C. beticola*, therefore, varied by a factor >2. The increase in the number of CLS lesions per leaf in time could be best described by an asymmetric sigmoid curve model (Figure S5). As an extended incubation period is considered to be characteristic for hemibiotroph pathogens [21], a large range in CLS incubation period is of particular interest for increasing our knowledge on the interactions between *C. beticola* and host plants within the period of asymptomatic disease development. Moreover, the high variation in IP is remarkable also in an epidemiologic context, as IP is a major component of partial resistance in host genotypes [39].

As *C. beticola* is a polycyclic pathogen able to reproduce within the vegetation period several times and to spread from initial infection sites to non-infected tissue and leaves, it was crucial to establish experimental conditions that enabled only a single period of leaf penetration. Otherwise, an increase in the number of disease symptoms may result from both a second generation of propagules (produced on the leaf surface) able to infect the leaf, and within-leaf spread of the pathogen by intercellular hyphae as observed for rust diseases, provided the incubation period is very long and leaf damage is limited [40]. Biotroph rust fungi often produce rather concentric rings of filial uredia surrounding the initial (=parental) uredium at a distance two to three times the diameter of uredia. Filial necrotic leaf spots would require latent hyphal growth for longer distances—4 to 8 mm—in the mesophyll, and would result in aggregations of CLS lesions in later stages of pathogenesis. As growth conditions in the greenhouse—RH <70%—and watering of plants without wetting the leaves prevented sporulation of *C. beticola* on primary CLS lesions, a second generation of leaf penetration and resulting symptoms was excluded. The continuing appearance of additional CLS lesions did not indicate to the formation of filial leaf spots, which would rather require waves of new lesions interrupted by time lags during periods of latent pathogen growth. The distribution of early CLS lesions revealed no spatial preferences, but homogenous and random patterns, and the increase in the number of CLS lesions also showed random patterns. Most of CLS lesions occurred in intercostal areas which have the highest portion in total leaf area. Nevertheless, variability of these host factors on a smaller scale cannot be excluded. Lesions appearing at the leaf margin often occurred in the first half of the IP range and remained small due to rapid desiccation of the thin plant tissue, whereas lesions on or near leaf veins had a prolonged growth period.

Differences in IP of individual CLS lesions may depend on the combination of differences in growth rate, virulence and aggressiveness of individual hyphae of *C. beticola* and/or variability in the local response (compatibility) of sugar beet leaf tissue to fungal development, i.e., pathogen detection and expression of defence mechanisms. For *C. beticola*, high genotypic diversity and low population differentiation were found between fields [41]. Genotypic pathogen diversity at the leaf level has been described for sugar beet [34,42] and table beet and Swiss chard [43]. A mixture of individual propagules differing in virulence and aggressiveness as well as in growth rate and toxin production is likely to contribute to IP differences in *C. beticola* on the leaf scale. Extended periods of optimum infection conditions had a positive effect on disease development irrespective of host susceptibility. Latecomers with a lower growth rate may be supported by a longer period optimal for infection, whereas differences in aggressiveness may be more important for within-leaf development. Formation of secondary hyphae and penetration of stomata did not depend on RH >90% which is in agreement with results that dry-wet cycles may even promote disease severity [11].

In addition to heterogeneity of *C. beticola* populations, spatial variation in the number of infectious propagules on the leaf surface may contribute to IP variability. Droplets of inoculum differ in the number of the very long, multicellular conidia which produce several germ tubes. An inoculum density of 2 × 10^4^ conidia per mL (and leaf) may result in 1 µL droplets of inoculum that contain 20 conidia. It is not known how many infectious conidia are required for successful leaf colonization; maybe 1 is sufficient, but multiple penetrations from one droplet of inoculum definitely multiplicate the pathogen biomass in leaf tissue and may reduce the incubation period of the developing CLS lesion.

As the length of both growth period on leaf surface and growth period in leaf tissue may contribute to IP differences, the experiments were generally conducted with a wetness period of 48 h; long enough to enable moderate disease levels, but relatively short in relation to IP ranging from 10 to 27 d p.i. Microscopic observations confirmed that germ tubes and secondary hyphae from a single conidium were able to penetrate sugar beet tissue more than once, but the maximum outreach of a conidium 100 µm in length was estimated to cover an area of 0.5 mm^2^, compared to an initial lesions size >1.2 mm^2^. Geostatistical analysis of CLS patterns early and later in pathogenesis did not support the idea of a large-scale endophytic spread of lesions from initial lesions. The formation of adjacent circular lesions by intercellular hyphal outgrowth from the margin of an existing lesion cannot be excluded; however, lateral expansions of CLS lesions as observed especially on older leaves became visible as lesion growth, not as another lesion.

IP was largely independent from (field) resistance of sugar beet cultivars—differences in CLS susceptibility by three grades (2 vs. 5) resulted in a 1-day difference in IP. This is in agreement with earlier reports [14]. Ontogenetic differences in resistance among leaves of plants were more important. Susceptibility to *C. beticola* infection increases from young to mature fully expanded and old leaves [44]. CLS lesions occurred first on old leaves, but the time of disease development (=increase in CLS number) often was limited because of leaf senescence which promoted tissue necrotisation (=lesion size) and death of the whole leaf.

The long IP of *C. beticola* of >10 d p.i. may also be explained by two days (with RH close to 100%) of epicuticular growth of germ tubes and secondary hyphae before tissue penetration via stomata and a subsequent period of quiescence without growth and nutrient uptake. However, growth and increase in *C. beticola* biomass in susceptible and resistant cultivars proved to be exponential after the sixth and third day p.i., respectively [33,45]. Tissue colonization and accumulation of hyphal mass were faster and stronger in susceptible varieties than in resistant ones. Exponential hyphal growth requires uptake of apoplastic nutrients in larger amounts.

The large within-leaf variation in IP seems to be in contrast to the role of *C. beticola* toxins reported in the formation of CLS lesions [26,27] and a switch from biotrophy to necrotrophy initiated by the pathogen. Cercosporin and beticolin toxins are only active in the light, and have no host specificity [27,46]. Targeted gene disruption in several *Cercospora* species emphasized its role as a virulence facilitator involved in necrosis induction [47,48]. Adaxial application of cercosporin on mature sugar beet leaves resulted in lesion formation after 5 days [29]. The ultrastructure of symptomatic tissue was very similar to lesions of leaves colonized by *C. beticola*, and included a mixture of damaged and non-damaged cells. Considering two to three days of epicuticular growth of germ tubes and the beginning of exponential hyphal growth 3 to 6 d after inoculation, the accumulated pathogen biomass and the amount of cercosporin produced by it may result in the formation of CLS lesions about 9 to 10 d after inoculation. Studies on the expression of the gene cluster for cercosporin biosynthese during pathogenesis are required to know the initiation of cercosporin exposure of sugar beet tissue—start with the colonization of tissue and slowly increase with *C. beticola* biomass, or as the switch to necrotrophy when fungal biomass has accumulated in the tissue in later stages. The lipophilic cercosporin readily penetrates plant leaves and leads to cellular damage within minutes of exposure [49]. As necrotic tissue is the basis of large-scale availability of nutrients for conidiation, cercosporin secretion has been speculated to facilitate cell wall breaching to enable the formation of conidiophores and conidia [45].

Coalescence of lesions reported to be the reason for heavy leaf damage [4,50] requires continuing hyphal growth and lesion expansion. In contrast, the appearance of additional lesions from a single infection period is likely to result in the filling of gaps between lesions with shorter IP. Actually, time and extent of growth of typical CLS lesions were very limited and the number of CLS lesions without immediate contact increased for a long period of time. IP variability of CLS lesions should also be relevant under field conditions, as disease severity may increase considerably even under prolonged periods without precipitation or dew, otherwise critical for the formation of conidia and a new generation of infections and symptoms.

### 4.2. Initial Lesion Area and Lesion Growth

The formation of typical CLS lesions involves a sequence of steps—tiny chlorosis, indention of leaf tissue, discoloration/necrotization of tissue, formation of a marginal ring varying in colour and size—and needs 24 to 48 h. Differences in early lesion formation and lesion type depending on host plant resistance have been characterized [51,52]. Average lesion size on sugar beet cultivars differing in CLS susceptibility was in the range reported earlier [5,24]; however, frequency distributions of lesion area and duration and extent of lesion growth exhibited large variability.

Latent colonization of living plant tissue requires the suppression of host resistance mechanisms and a longer IP was expected to result in extended fungal growth and, hence, larger initial lesion area. However, the initial CLS lesion area on leaves of four cultivars did not increase with IP, except for a rather old and highly susceptible leaf. Individual propagules of *C. beticola* differ in growth rate (and aggressiveness) and the amount of fungal biomass (and toxins) produced is likely to trigger the visible necrotization of host tissue, rather than developmental time. Formation of CLS lesions involves the near-simultaneous collapse of cells in an area covering some mm^2^, commonly followed by the formation of a characteristic reddish-brown ring sharply delimiting the lesion from green leaf tissue [5]. After appearance, lesion growth is rather limited and the increase in total necrotic leaf area is due primarily to an increase in the number of lesions.

A decline in compatibility between host and pathogen during hyphal growth in plant tissue may be due to the formation of new structures, a shift in the release of effectors (enzymes, etc.) and continuous toxin secretion by the pathogen, or due to the kick-in of resistance mechanisms of the plant. The formation of a ring of reddish-brown and tissue with fortified cell walls that delimits the lesion sharply from surrounding green tissue indicates that lesion formation may be initiated rather by plant tissue than by fungal hyphae. Strengthened cell walls at the lesions’ margin block fungal activities (growth and diffusion of toxins). Incrustation of phenolics significantly increase autofluorescence of plant cell walls, especially ferulic acid derivatives increase wall stability and coherence of cells in response to abiotic and biotic stressors [53].

The very similar initial lesion area suggests symptom development depending on fungal biomass and/or cercosporin amount experienced by sugar beet cells. Fungal biomass, however, may also be a trigger for a shift in pathogen metabolism. A process similar to quorum sensing or quorum signalling described for cell-to-cell communication enabling bacteria to detect and respond to cell population density could provide the information that fungal biomass has exceeded a threshold sufficient for initiating conidiation. Formation of the very long conidia requires huge amounts of nutrients which are not available from non-haustorial nutrient uptake from the apoplast, but depend on disintegration of many host cells.

Provided lesion development is initiated by *C. beticola* via toxins and effectors, the question arises as to why the majority of CLS lesions on young leaves do not expand after their appearance. Release of the pathogen’s CWDEs arsenal or toxins may be in response to host cell death. In other hemibiotrophic interactions, the pathogen responds to host plant activities. The loss of integrity of the plant-derived extra-invasive hyphal membrane enclosing *Magnaporthe oryzae* invasive hyphae preceded shrinkage and eventual rupture of the rice vacuole which coincided with host cell death [54]. In *Colletotrichum* species, pre-invasion perception of plant-derived signals substantially reprograms gene expression [55].

Lesion growth on partially resistant host genotypes visualized the dynamics of pathogen expansion and plant response. Inhibition of *C. beticola* hyphal growth by host defence mechanism(s) was faster and, therefore, more effective in resistant than in susceptible genotypes. The period of lesion growth in young tissue of susceptible cultivars was limited in time. Lesion growth was strong and persistent only for old leaves exhibiting a low level of resistance. For most host genotypes, a large portion of CLS lesions developed within 24 to 48 h from tiny yellow initial spots to lesions surrounded by a darker margin, but did not increase in size afterwards. The relative growth rate of expanding lesions decreased (exponentially), and only a small portion of lesions continued to expand for a longer period of time. It is not clear whether subareas of leaves differ in the effectiveness of resistance or the variation is random and follows a normal distribution. The smaller area of CLS lesions on resistant cultivars 7 d after appearance confirmed earlier results where the effect of CLS resistance was significant only between the most resistant and the most susceptible cultivar, whereas the effect of environmental conditions was always significant [24].

### 4.3. Tissue Colonization

Intercellular hyphae of *C. beticola* in the early, asymptomatic phase were much wider (3–6 µm) than epicuticular germ tubes and secondary hyphae (1.4–2.1 µm; see [5]) and intercellular hyphae produced after the appearance of CLS lesions (2–3 µm). Dimorphism is described for yeast-like fungi, e.g., Candida *albicans* and smut fungi changing from budding of circular cells to filamentous growth initiated by environmental clues [56,57]. For a variety of fungal pathogens, the shift between hyphae and yeast is critical for pathogenesis (e.g., *C. albicans* and *Ceratocystis ulmi*; [58]). Dimorphism of hyphae outside and within plant tissue has been described for the hemibiotrophs *M. oryzae* and *Colletotrichum* spp. producing intracellular bulbous invasive hyphae or haustorium-like hyphae and thinner necrotrophic hyphae [59,60,61]), as well as for *Venturia inaequalis* that produces runner hyphae and wide, but very thin fan-shaped hyphae discussed to be effective in nutrient uptake [62]. Wide hyphae may be understood as indicator of biotrophy, i.e., the pathogen benefits from an increased interface with plant cells which guarantee homoeostasis of environmental conditions compared to necrotrophic and saprotrophic hyphae exposed to varying and often unfavourable conditions, e.g., low RH and defence mechanisms of the host.

External signals like changes in metabolite levels within host cells harbouring biotrophic fungal structures, e.g., depletion of sugars, nitrogen starvation, production of toxins, and massive release of CWDEs have been discussed as triggers for the switch of the pathogen from biotrophy to necrotrophy [63]. At 1 h after wounding, colonies of *Trichoderma viride* produced new, thinner hyphal tips which exclusively produced phialides and mature spores within 48 h [58]. Stress conditions induce the change in hyphal morphology that is mediated by known genes [64]. In case of *C. beticola*, thin hyphae have a surface/volume ratio optimal for less favourable environmental conditions in necrotic tissue, as well as on the plant surface.

The fluid of the extracellular space outside the plasma membrane of plant cells—cell wall, xylem, and any space between cells—contains water, sugars, amino acids, cell wall modifying enzymes, growth regulators, stress-related proteins, and extracellular vesicles that carry defence-related proteins and small RNAs [65]. The amount and composition of its nutrients may be suitable for continuous intercellular fungal growth.

The occurrence of numerous protuberances of sugar beet cells around CLS lesions indicated to an effector (toxin?) activity of *C. beticola* probably targeting the nutrient availability in the leaf apoplast. Protuberances of leaf cells of infected and cercosporin-treated sugar beet leaves have been reported by [29]. Protuberances of pectic material have been reported from sugar beet root parenchyma and marginal areas of rust lesions of wheat [29]. They may be indicators of membrane damage and the leakage of cellular material from affected cells. As this phenomenon was observed also in non-colonized tissue surrounding CLS lesions, the effect of cercosprin on plant cell integrity may exceed the lesion area.

After appearance of CLS lesions, the period of lesion growth was short, except for older leaves, which had reduced resistance because of senescence. Microscopic observations in leaf tissue of susceptible cultivars gave evidence for continuing attempts of hyphal (diameter < 3 µm) growth outside the dark marginal ring of CLS lesions. These hyphae of *C. beticola* were not able to switch from necrotrophy (=thin hypha) to biotrophy (diameter > 3 µm); biotrophic hyphae may develop only from conidia, epicuticular germ tubes and appressoria and/or in the absence of necrotic plant cells.

Growth pattern of *C. beticola* hyphae was intercellular between mesophyll cells, even at the stage of pseudostroma formation, the prerequisite for sporulation. Rare penetration attempts by hyphae with a diameter of 2–3 µm caused cell death whereas mesophyll cells in the centre of CLS lesions in contact with wide hyphae whether non-melanized or melanized, exhibited no visible damage. As cercosporin damages plant cells by peroxidation of membrane lipids and, thus, causes cell leakiness and increased nutrient availability in the apoplast, it is not necessary for *C. beticola* to destroy cell walls before nutrient requirements increase with sporulation. Direct penetration of host cells and intracellular hyphal growth of *C. beticola* is typical for sporulating lesions with highly degraded and necrotic tissue [9]. In this study, without the induction of conidiation, intracellular hyphal growth was restricted to tissue of the brownish ring.

The term hemibiotroph has been used for different lifestyles of fungi [59,66]: (I) members of the genus *Colletotrichum* and *M. oryzae* form temporally separate and non-overlapping biotrophic and necrotrophic structures in the plant [67]; and (II) species such as *Fulvia fulva*, *Z. tritici* and *Pyrenopeziza brassicae* have an extended (4–14 day) asymptomatic phase assumed to correspond to the acquisition of nutrient from living cells without having differentiated infection hyphae; this biotrophic phase is followed by a switch to necrotrophy and reproduction associated with increasing plant tissue damage.

The definition of hemibiotrophy is variable and criteria to justify this classification are rare [63]. Precigout et al. [21] listed 15 species as hemibiotrophs in their study on the relation between latent period and trophic type. Not included in this study, *M. grisea*, *Colletotrichum lupine*, *Oculimacula yallundae*, and *Moniliophthora roreri* have also been reported as hemibiotrophs [54,68,69,70]. However, growth and nutrient acquisition by of *Z. tritici,* one of the most frequently studied hemibiotroph, has also been named a latent necrotroph [71]. The definition of hemibiotrophs has been relaxed ‘as pathogens displaying a two-stage infection cycle characterized by contrasting interactions with the host: an asymptomatic colonization in the living host tissues followed by a necrotrophic stage with sustained asexual reproduction’ [21].

Often, the biotrophy of plant pathogens is associated with the formation of haustoria (or other specialized fungal structures in close contact with host cells) for nutrient uptake from living cells. In case of nutrient acquisition without specialized hyphae, classification of the fungal lifestyle as biotrophy is problematic, because nutrient uptake may be very similar to growth on artificial media (and saprotrophy). A criterion for differentiation may be the production of effectors by the colonizing fungus, which should improve the nutrient situation in the apoplast for biotrophs, whereas others have to be content with the nutrients present in the apoplast without colonization.

## 5. Conclusions

*C. beticola* on sugar beet has been classified as a hemibiotroph pathogen, although it produces several toxins involved in pathogenesis. A biotrophic stage of *C. beticola* is concluded from the observation of a long incubation period. Biotrophy may be used to characterize the colonization of plant tissue without causing disease symptoms; in powdery and downy mildews and rusts, fungal structures (spores and sporangiophores) on the leaf surface are the typical disease symptoms. *C. beticola* neither produce haustoria nor haustoria-like structures within host cells, but thicker intercellular hyphae as long as necrotic plant cells are lacking, and thinner and melanized hyphae after lesion appearance and induces protuberances of host cells. Secretion of cercosporin (and other substances) causes membrane damage and leakiness of host cells that may increase nutrient availability in the apoplast, and ultimately results in cell death. Information on the time course of in planta expression of the cercosporin biosynthesis gene cluster and of the vitality of plant cells during asymptomatic fungal colonization would be highly welcome. An increase in the secretion of toxins—and hence an increased activity of accumulated toxins—is also suitable to explain (limited) growth and the eventual appearance of CLS lesions characteristic for necrotrophy. Despite of having a long and highly variable incubation period, evidence for a true biotroph phase of *C. beticola*—i.e., nutrient uptake from living plant tissue—is lacking.

## Figures and Tables

**Table 1:** Details on the appearance and development of CLS lesions on individual leaves of four sugar beet cultivars varying in CLS susceptibility.

Parameter	Sugar Beet Genotype			
	Carsta (2) ^1^	Debora (5)	Emilia (5)	Pauletta (4)
Total leaf area [mm^2^]	6.486	6.867	5.944	9.455
Initial CLS lesion(s):				
- First appearance [d p.i.]	11	10	10	10
- No. of lesions	8	8	1	72
- Lesion area [mm^2^] ^2^	1.18	1.13	1.11	1.27
- Total lesion area [mm^2^]	8.8	6.5	1.1	115
CLS lesions 17 d p.i.:				
- No. of lesions	170	43	95	661
- Lesion area [mm^2^] ^2^	6.14	14.72	5.59	14.55
- Total lesion area [mm^2^]	759	494	628	2.922
- Correlation time × area (r)	0.40 (*p* ≤ 0.001)	0.40 (*p* ≤ 0.01)	0.21 (*p* ≤ 0.05)	0.40 (*p* ≤ 0.001)
Mean growth duration [d] ^3^	5.8 ± 0.2 (n = 26)	4.9 ± 0.5 (n = 27)	2.5 ± 0.3 (n = 94)	2.8 ± 0.2 (n = 231)

^1^ CLS susceptibility on a scale 1(low) to 9 (high); ^2^ median; ^3^ mean ± SEM.

**Table 2:** Characterization of the spatial distribution of CLS lesions on individual leaves of four sugar beet genotypes differing in CLS susceptibility.

Parameter	Sugar Beet Genotype			
	Carsta	Debora	Emilia	Pauletta
No. of grids	23	25	20	27
First CLS appearance [d p.i.]	11	10	10	10
- No. of lesions per leaf	8	8	1	72
- No. of lesions per grid	0.35 ± 0.10 ^1^	0.32 ± 0.09	0.05 ± 0.05	2.67 ± 0.40
+ Variance-to-mean ratio	0.65	0.68	0.95	1.61
CLS lesions, 17 d p.i.:	170	43	95	661
- No. of lesions per grid	7.39 ± 0.491	1.72 ± 0.26	4.75 ± 0.53	24.48 ± 1.60
+ Variance-to-mean ratio	0.76	1.00	1.20	2.83
- Increase in no. of lesions	162	35	94	589
- Increase no. lesion per grid	7.04 ± 0.48 ^1^	1.40 ± 0.26	4.70 ± 0.52	21.81 ± 1.51
+ Variance-to-mean ratio	0.75	1.20	1.17	2.84
+ Grids without lesion	6.9	2.1	4.6	19.0
+ Grids with lesion(s)	7.3	1.1	6.0	22.2

^1^ Mean ± SEM.

## Data Availability

The raw data supporting the conclusions of this article will be made available by the authors on request.

## Supplementary Materials

The following supporting information can be downloaded at: https://www.mdpi.com/article/10.3390/jof11030211/s1. Figure S1: Appearance and development of Cercospora leaf spots on a leaf of sugar beet cv. Carsta in the period 10 to 17 d p.i.; Figure S2: Appearance and development of Cercospora leaf spots on a leaf of sugar beet cv. Emilia in the period 10 to 27 d p.i.; Figure S3: Relative growth rate of CLS lesions during pathogenesis on cv. Carsta (left) and cv. Emilia (right), respectively; Figure S4: Fortification of sugar beet cell walls at the transition from necrotic margin to healthy tissue. Autofluorescence of cell walls of cv. Britta (left; additional staining with diethanol) and Pauletta (right); Figure S5: Epicuticular growth of *C. beticola* germ tubes on sugar beet leaves; Figure S6: Curve fitting of the increase in the number of CLS lesions per leaf during pathogenesis (cv. Emilia, TableCurve 2D).
