# Supplementary material for: Intra-Leaf Variability of Incubation Period Sheds New Light on the Lifestyle of Cercospora beticola in Sugar Beets
Source: J Fungi (Basel). 2025 Mar 9;11(3):211. doi: 10.3390/jof11030211 (PMC11943282; doi:10.3390/jof11030211)
Supplement: Supplementary file 1 [file jof-11-00211-s001.zip › jof-3508412-supplementary.pdf]

Supplementary Material

Intra-Leaf Variability of Incubation Period Sheds New Light on the Lifestyle of *Cercospora beticola* in Sugar Beets

Erich-Christian Oerke* and Ulrike Steiner
Institute of Crop Science and Resource Conservation - Plant Pathology, Nussallee 9, D-53115 Bonn, Germany

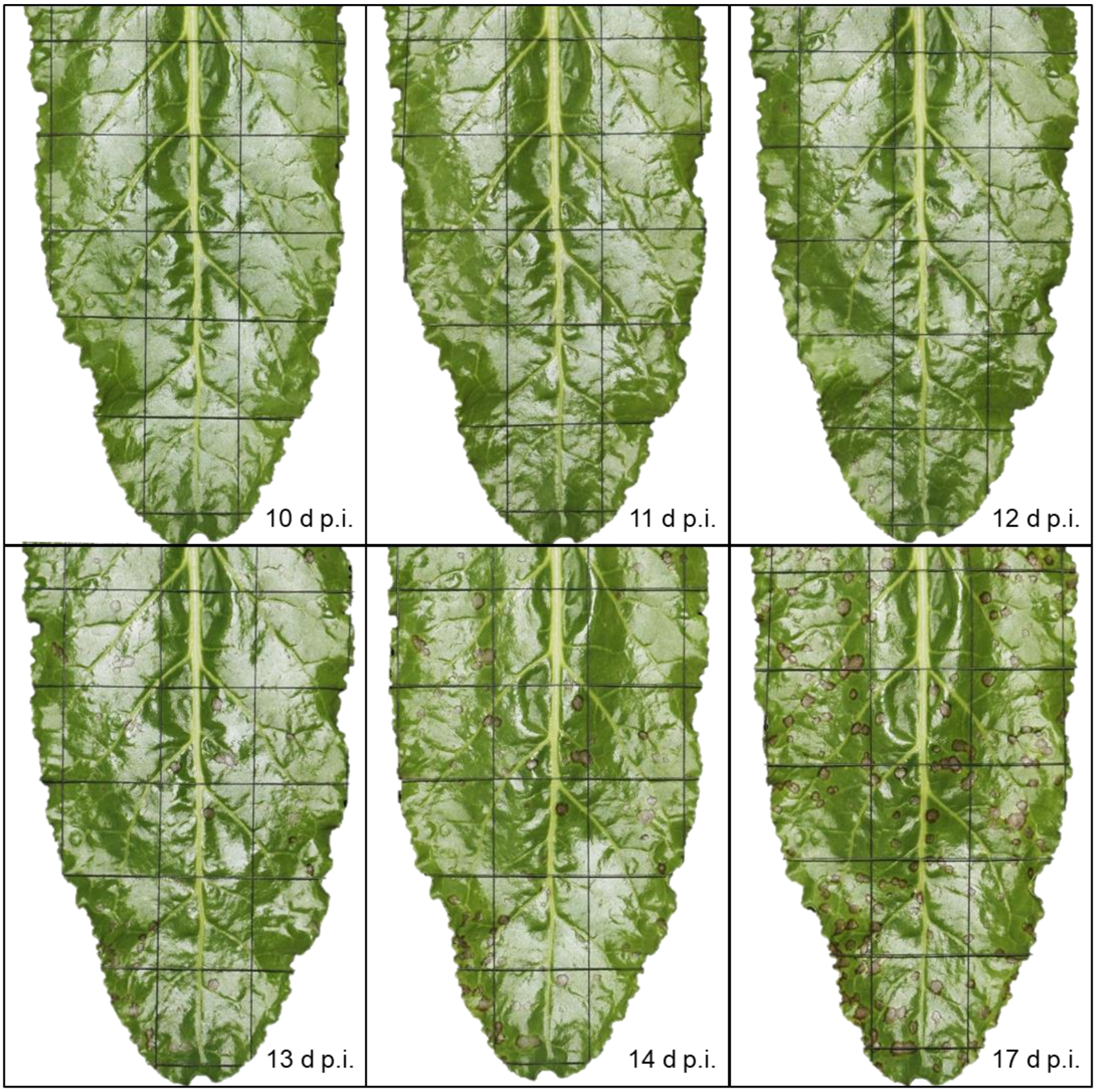

Figure S1. Appearance and development of Cercospora leaf spots on a leaf of sugar beet cv. Carsta in the period 10 to 17 d p.i..

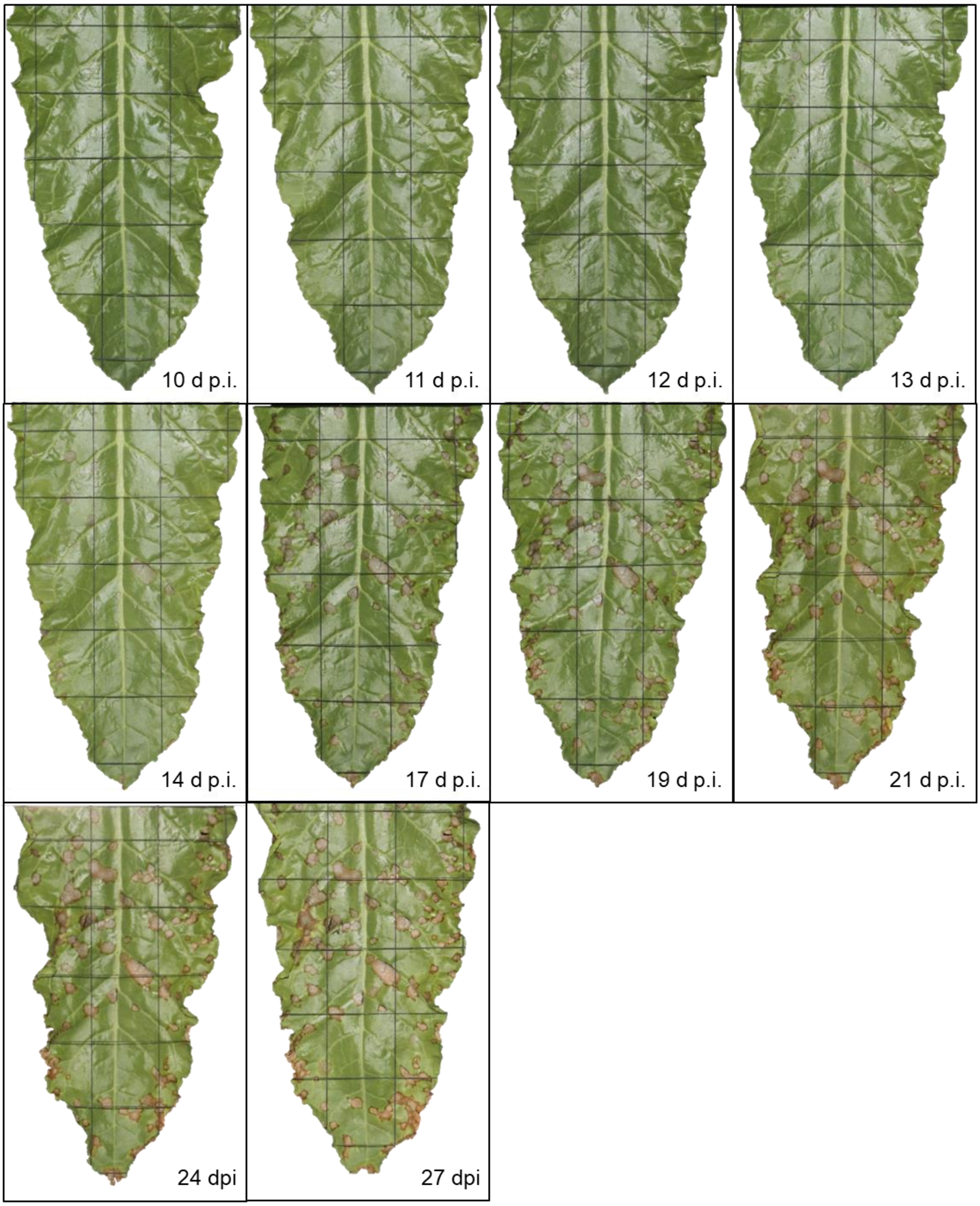

Figure S2. Appearance and development of Cercospora leaf spots on a leaf of sugar beet cv. Emilia in the period 10 to 27 d p.i..

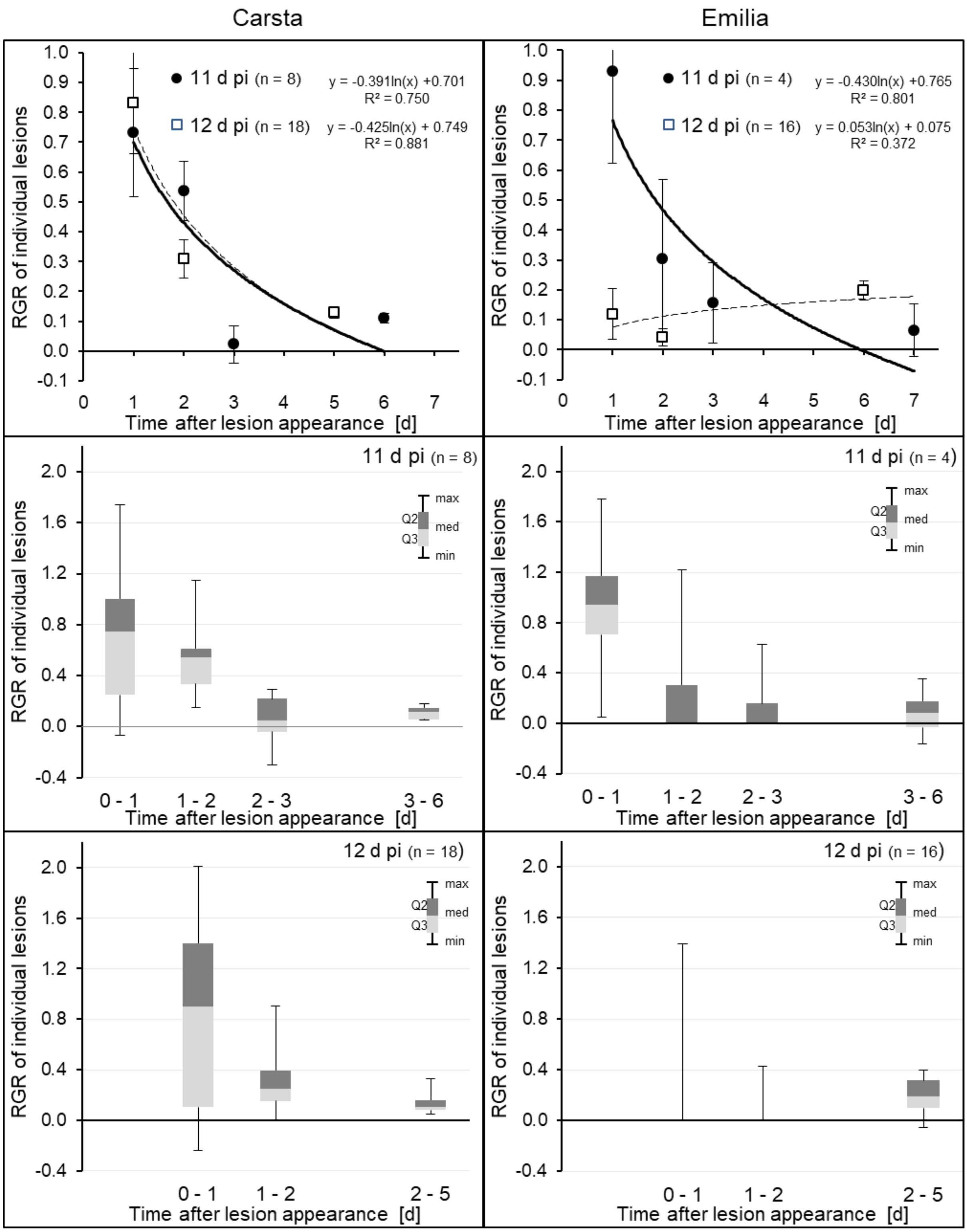

Figure S3. Relative growth rate of CLS lesions during pathogenesis on cv. Carsta (left) and cv. Emilia (right), respectively.

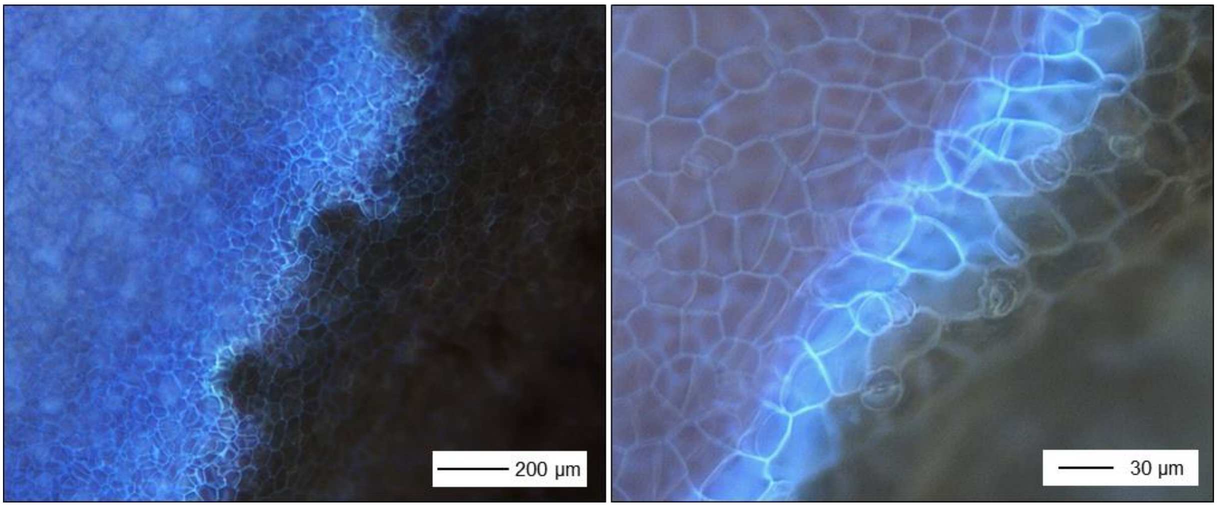

Figure S4. Fortification of sugar beet cell walls at the transition from necrotic margin to healthy tissue. Autofluorescence of cell walls of cv. Britta (left; additional staining with diethanol) and Pauletta (right).

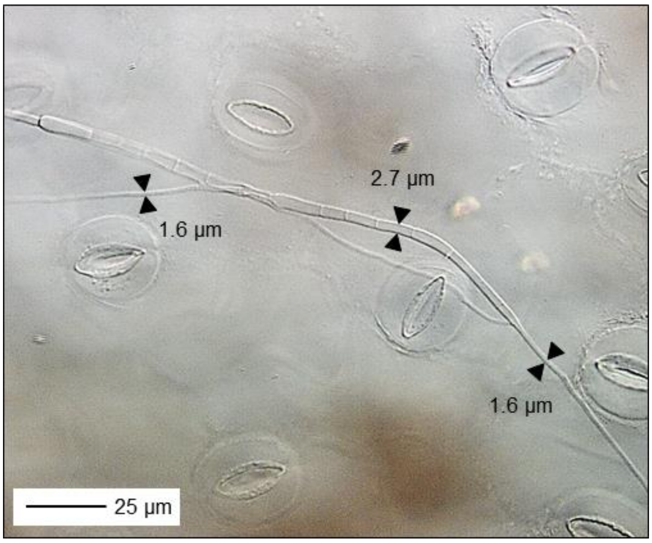

Figure S5. Epicuticular growth of *C. beticola* germ tubes on sugar beet leaves.

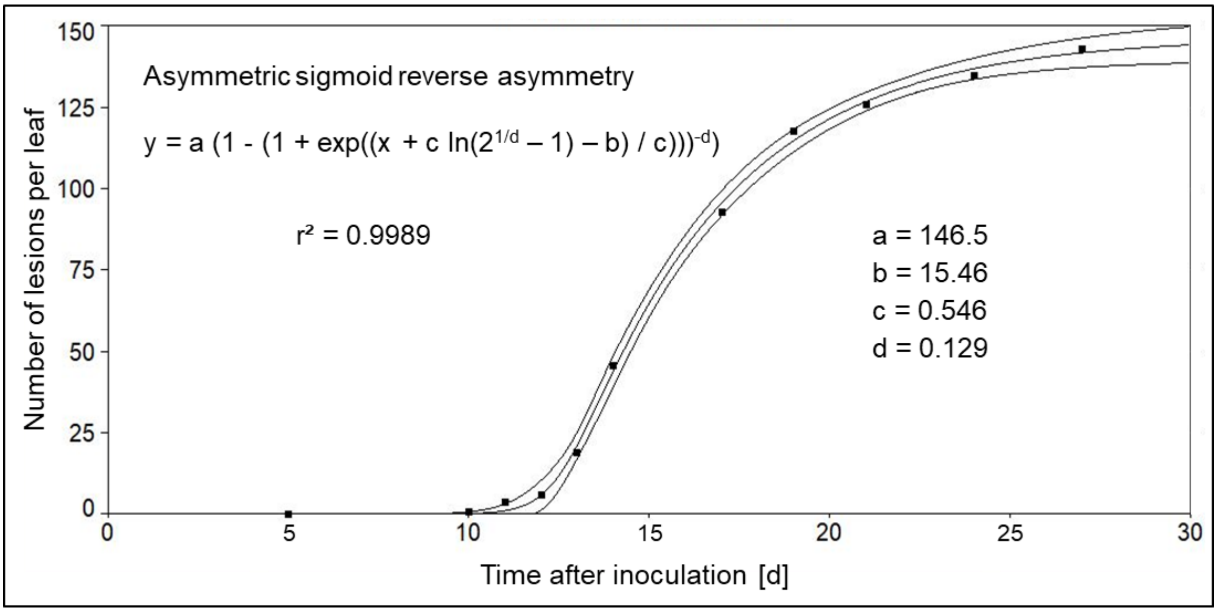

Figure S6. Curve fitting of the increase in the number of CLS lesions per leaf during pathogenesis (cv. Emilia, TableCurve 2D).
